# Vitamin D Fortification of Fluid Milk Products and Their Contribution to Vitamin D Intake and Vitamin D Status in Observational Studies—A Review

**DOI:** 10.3390/nu10081054

**Published:** 2018-08-09

**Authors:** Suvi T. Itkonen, Maijaliisa Erkkola, Christel J. E. Lamberg-Allardt

**Affiliations:** Department of Food and Nutrition, P.O. Box 66, 00014 University of Helsinki, 00790 Helsinki, Finland; maijaliisa.erkkola@helsinki.fi (M.E.); christel.lamberg-allardt@helsinki.fi (C.J.E.L.-A.)

**Keywords:** dairy, vitamin D, vitamin D-fortified milk, vitamin D intake, vitamin D fortification, 25-hydroxyvitamin D

## Abstract

Fluid milk products are systematically, either mandatorily or voluntarily, fortified with vitamin D in some countries but their overall contribution to vitamin D intake and status worldwide is not fully understood. We searched the PubMed database to evaluate the contribution of vitamin D-fortified fluid milk products (regular milk and fermented products) to vitamin D intake and serum or plasma 25-hydroxyvitamin D (25(OH)D) status in observational studies during 1993–2017. Twenty studies provided data on 25(OH)D status (*n* = 19,744), and 22 provided data on vitamin D intake (*n* = 99,023). Studies showed positive associations between the consumption of vitamin D-fortified milk and 25(OH)D status in different population groups. In countries with a national vitamin D fortification policy covering various fluid milk products (Finland, Canada, United States), milk products contributed 28–63% to vitamin D intake, while in countries without a fortification policy, or when the fortification covered only some dairy products (Sweden, Norway), the contribution was much lower or negligible. To conclude, based on the reviewed observational studies, vitamin D-fortified fluid milk products contribute to vitamin D intake and 25(OH)D status. However, their impact on vitamin D intake at the population level depends on whether vitamin D fortification is systematic and policy-based.

## 1. Introduction

Vitamin D plays an important role in bone health, being necessary for calcium absorption [1]. Low vitamin D status in terms of low serum 25-hydroxyvitamin D (S-25(OH)D) concentration has also been linked to the increased risk of some common chronic diseases, such as type 2 diabetes or cardiovascular disease [2]. In Northern latitudes, especially in the wintertime, ultraviolet B (UVB) radiation is too low for dermal synthesis of vitamin D [3]. As there are only a few natural vitamin D-rich foods, such as fish, egg yolk, and some wild mushrooms [1], some countries, particularly populations at high latitudes, have initiated national policies of fortifying certain foods with vitamin D to prevent vitamin D deficiency. Usually these vitamin D-fortified products are low-fat milk, fat spreads, breakfast cereals, and certain baby foods [4,5]. To better cover different population groups with differing food habits, a wider vitamin D fortification of different products instead of concentrating on only a few staple foods has been suggested [5].

To our knowledge, a portion of milk products are systematically, either mandatorily or voluntarily, fortified with vitamin D only in Finland, Norway, Sweden, Canada, and United States (Table 1) [6,7,8,9,10,11,12]. In Finland, the recommended fortification level of all fluid milks except some organic products is currently 1 µg/100 g, but some products with a concentration of 2 µg/100 g are available on the market [6,7]. The fortification is voluntary, but all manufacturers unanimously follow the recommendations. In Norway, only one type of milk is recommended to be fortified with vitamin D at a concentration of 0.4 µg/100 g [8]. Sweden recently doubled the fortification levels of fluid milks to 1 µg/100 g and extended the mandatory fortification to cover all fluid milk products with <3% fat [9,10]. Health Canada has also proposed increasing the mandatory vitamin D fortification of fluid milks from around 1 µg/100 g to 2 µg/100 g as a consequence of the inadequate vitamin D intake among the population [11]. In the United States, fluid milks can be fortified with vitamin D by around 1 µg/100 g; the fortification is not mandate at the federal level, but most states mandate fortification [12]. In other countries, such as United Kingdom, Ireland, Spain, and Australia, the fortification is not systematic, but there is a varying number of vitamin D-fortified milk products available [13,14,15,16,17]. However, data on their proportion to the total amount of dairy products in different countries is not easily accessed due to fluctuations in the market. This causes a knowledge gap on the prevalence of vitamin D fortified fluid milks and their contribution to vitamin D intake worldwide.

In the latest updated systematic review and meta-analysis on the effects of vitamin D fortification in randomised controlled trials (RCT), 12 of the 16 included studies used different milk products, such as fluid milk or milk powder, as a carrier of vitamin D [18]. Four of these studies used vitamin D-fortified milk and two used vitamin D-fortified yoghurt drinks. All of the studies showed the efficacy of the studied milk products to increase the S-25(OH)D concentration or decrease the decline in S-25(OH)D status during the wintertime relative to the control group [19,20,21,22,23,24]. Further, in Finland, the vitamin D fortification of fluid milks has been shown to improve the S-25(OH)D status independently among regular milk users after extensive changes in the national vitamin D fortification policy in an 11-year follow-up study [25].

The aim of this review was to investigate the contribution of vitamin D-fortified fluid milk products (regular milk and fermented products, such as sour milk and yoghurt) (i) to vitamin D intake; and (ii) to vitamin D status (25(OH)D concentration in plasma or serum in observational studies with a special focus on differences possibly caused by different vitamin D fortification policies.

## 2. Materials and Methods

### 2.1. Data Sources and Search Strategy

The literature search was done in the PubMed database at the end of December 2017. The search terms were the following combination of keywords: “vitamin D” [MeSH Terms] OR “vitamin D” [All Fields] OR “ergocalciferols” [MeSH Terms] OR “ergocalciferols” [All Fields]) AND (dairy [All Fields] OR (“milk, human” [MeSH Terms] OR (“milk” [All Fields] AND “human” [All Fields]) OR “human milk” [All Fields] OR “milk” [All Fields] OR “milk” [MeSH Terms])) AND (fortification [All Fields] OR fortified [All Fields]”. We limited the search to articles that had the search terms in their title, abstract or among keywords. The data search was restricted to the last 25 years from 1993 to 2017.

### 2.2. Eligibility and Study Selection

Two independent authors reviewed the titles and abstracts of all identified studies and selected observational studies that reported either vitamin D intake or 25(OH)D status in plasma or serum for full-text screening. Among the full-texts, the eligibility of the articles was screened using the following exclusion criteria: full-text in a language other than English, studies with disease outcomes, participants/patient groups with diagnosed diseases, participants aged less than one year, studies in which the contribution of *growing up milks* (special products marketed for 1 to 3-year-olds) could not be separated from that of other fluid milks, RCTs, and reviews. In addition, nationally representative study reports in local languages other than English were searched to cover vitamin D intake data from all countries with vitamin D fortification policy of fluid milks.

### 2.3. Data Extraction

The following information was extracted from eligible studies: first author’s name, publication year, country, number and age range of subjects, dietary assessment method, total and/or dietary vitamin D intake (vitamin D intake studies), vitamin D intake from milk (vitamin D intake studies), contribution of milk to vitamin D intake (vitamin D intake studies), latitude (25(OH)D studies), season that blood was drawn (25(OH)D studies), 25(OH)D assay method and quality control of assay (25(OH)D studies), and 25(OH)D concentrations (25(OH)D studies). The results were stratified by the population groups as follows: “children and adolescents” (vitamin D and 25(OH)D studies), “pregnant women and mother-child pairs” (25(OH)D studies), and “adults, elderly, and all age groups” (vitamin D and 25(OH)D studies). In addition, the results were reported by country. In some cases, the results were stratified by supplement use or other factors, depending on the original study design. In 25(OH)D studies, the role of vitamin D-fortified milk on vitamin D status was examined as a determinant of vitamin D status or as a comparison of the 25(OH)D status between low or non-users of vitamin D-fortified milk and more frequent users. If the contribution of milk to total vitamin D intake was not provided, it was calculated from the reported total intake and vice versa. International units were converted to micrograms, and 25(OH)D concentrations in ng/ml were converted to nmol/L. In this study, we referred to the Institute of Medicine threshold for S-25(OH)D status, where ≤30 nmol/L is vitamin D deficient, 30–49.9 nmol/L is insufficient, and ≥50 nmol/L is sufficient [26].

## 3. Results

**Figure 1:**
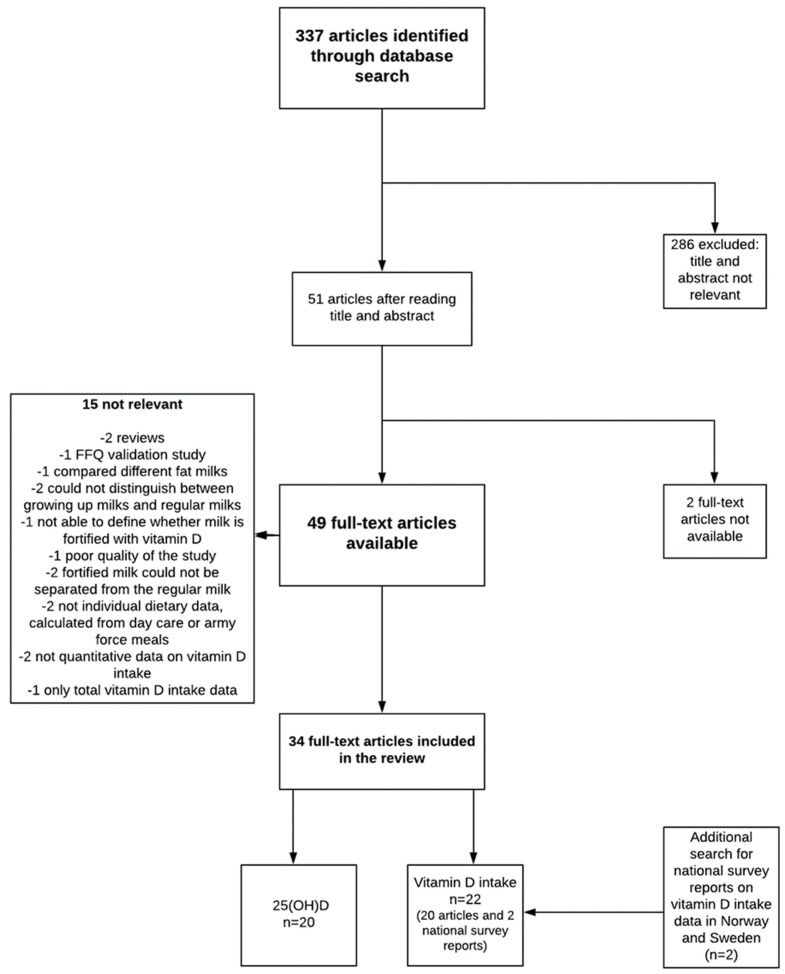
Literature search and study selection process.

Figure 1 shows the literature search and study selection process. We found 337 articles that were published between 1993 and 2017, and their titles and abstracts were scanned. Fifty-one full-text review papers were selected. Of these, two were unavailable and the corresponding author could not be reached, and 15 were not relevant to the research questions. Thus, 34 papers were included in the review process. Of these, 20 provided data on the 25(OH)D status, and 20 provided data on vitamin D intake. Additionally, intake data in national surveys covering countries with a fluid milk vitamin D fortification policy that were not covered in the PubMed search (Norway, Sweden) were explored. One Norwegian [27] and one Swedish report [28] in local language were found and were included to provide data on vitamin D intake and the contribution of milk in those countries. Nationally representative data from other countries with a vitamin D fortification policy were already found in the literature search.

### 3.1. Contribution of Vitamin D-Fortified Milk to Vitamin D Intakes

For this review, 22 observational studies reported data on the contribution of vitamin D-fortified milk to vitamin D intake including 99,023 subjects (Table 2). Data from the following countries were provided: United States (6 studies), Canada (4), Finland (4), Ireland (2), Australia (1), Norway (1), Spain (1), Sweden (1), and United Kingdom (1). Additionally, one study provided data from both the United States and Canada. Various methods to assess vitamin D intake were used: food records (8 studies), 24 h recalls (7), food frequency questionnaires (FFQ) (4), one-week diet history (1), household food diary (1), and both FFQ and food records (1).

#### 3.1.1. Children and Adolescents

In the studies of Irish and British children and adolescents, the total vitamin D intakes were 2.8–3.5 µg/day and dietary intakes were 1.6–2.6 µg/day [13,14,15]. Fortified milks provided 0.4 µg/day or less vitamin D [13,14,15]. It is notable that the consumption of vitamin D-fortified milk was not common; in the study of Black et al. [13], only 4–5% of subjects consumed vitamin D-fortified milk. In contrast, in countries with policy-based vitamin D fortification, i.e., in the United States, Canada, and Finland, the mean dietary vitamin D intakes in children were 4.4–5.9 µg/day, and 2.3–3.3 µg/day of that originated from milk products, covering more than half of the total dietary intake [29,30,31,32].

#### 3.1.2. Adults and the Elderly, and Studies Including All Age Groups

In Spain and Australia, some of the fluid milks on the market are fortified with vitamin D and studies conducted in these countries among the adult population showed that the contribution of milk to total vitamin D intake was 15–18%, with total intakes being 3.5 and 4.4 µg/day, respectively [16,17]. In a Canadian population-based study among adults, the total vitamin D intake was shown to be 5.6 µg/day among females and 4.8 µg/day among males, and milk contributed 48% of the total vitamin D intake among females and 63% among males [33]. In other large American and Canadian population-based studies covering all age groups, 1.9–2.9 µg of vitamin D ingested per day originated from milk, while mean total vitamin D intakes ranged from 4.2 to 9.8 µg/day and dietary intakes from 3.9 to 7.0 µg/day, milk contributing 44–49% of the vitamin D intake [33,34,35,36,37]. In a smaller study carried out among the adult population in the United States as well in a Canadian study on Inuit and Inuvialuit women, vitamin D intakes were similar to those found in the larger studies; however, the contribution of milk to vitamin D intake was slightly lower, 31–43% [38,39]. In line with the newer studies, in two studies carried out among elderly people in the United States that were published in the 1990s, half of the vitamin D intake originated from milk [40,41]. The recent representative population-based study in Finland [25] showed that 34% of dietary vitamin D intake originated from vitamin D-fortified fluid milk products which is similar to the proportion observed in the latest National FINDIET Study—28–39%, varying between age and sex groups [42]. Dietary vitamin D intakes in the study of Jääskeläinen et al. were the highest among all of the studies included in this review: 14 µg/day among men and 12 µg/day among women [25]. Data on the contributions of milk to vitamin D intake in other Nordic European countries following the implementation of a national vitamin D fortification policy have also been provided. The latest Norwegian national dietary survey reported that extra-skimmed milk, the only vitamin D-fortified milk in Norway, provided 4% of dietary vitamin D intake, with the mean dietary vitamin D intake being 6 µg/day [27]. Despite the wider milk fortification policy in Sweden, only 12% of dietary vitamin D intake originated from milk products in the Swedish national survey, with the mean dietary vitamin D intake being 7 µg/day [28].

### 3.2. Associations Between Consumption of Vitamin D-Fortified Milk and 25(OH)D Status

Twenty observational studies included in this review investigated associations between the consumption of vitamin D-fortified milk and 25(OH)D status (*n* = 19,744) (Table 3). Data from the following countries were provided: United States (5 studies), Canada (4), Finland (3), Sweden (2), Egypt (1), Ireland (1), Jordan (1), Norway (1), Spain (1), and Thailand (1). Various methods were used to assess 25(OH)D concentrations: different immunoassays (13 studies), LC-MS/MS (5) and competitive binding assays (2). Milk consumption was assessed by either a questionnaire (8 studies), FFQ (5), food records (4), 24 h recall (1), one-week diet history (1) or by both FFQ and food records (1).

#### 3.2.1. Children and Adolescents

Among Egyptian children aged 9–11 years (*n* = 200), those who consumed vitamin D-fortified milk less than once a day had a significantly higher risk of vitamin D insufficiency (S-25(OH)D < 50 nmol/L) than those who consumed more milk [43]. In Jordan, children who consumed vitamin D-fortified fresh milk had higher S-25(OH)D concentrations than those who consumed unfortified milk (53 nmol/L vs. 43 nmol/L) (*n* = 93) [44]. These two studies did not provide data on the amounts of consumed milk. In Finland, higher consumption of vitamin D-fortified milk was associated with higher S-25(OH)D concentrations among children aged 6–8 years (*n* = 374) [31]. Children who drank at least 450 g/day of vitamin D-fortified milk had a 72–74% lower risk of having S-25(OH)D below 50 nmol/L than those who drank less than 300 g/day (adjusted for age and sex). However, another study on 10-year-old Finnish children (*n* = 171) found no association between vitamin D-fortified milk consumption frequency and S-25(OH)D status, but among those children with a history of cow’s milk allergy (an indicator of milk avoidance), consumption of vitamin D-fortified milk as well as S-25(OH)D concentrations were lower than among their peers without allergy history [45]. Nevertheless, vitamin D supplement use was very common in this population (60% daily users), and thus was one important determinant of their vitamin D status [45].

A Canadian study in 1–6-year-old children (*n* = 2468) showed that children who drank only non-cow’s milk (i.e. vegetable-based milk alternatives or goat’s milk) were more than two-fold likely to have an S-25(OH)D concentration <50 nmol/L relative to children who drank cow’s milk, which is mandatorily fortified in Canada (odds ratio 2.7, 95% CI 1.6–4.7) [46]. In another sample of Canadian children aged 8–11 years with a daily mean of 1.3 vitamin D-fortified milk servings (*n* = 159), one daily serving of milk contributed to a 2.9 nmol/L increase in plasma 25(OH)D concentration [30]. Further, among 2270 children aged 3–18 years in Canada, those consuming vitamin D-fortified milk daily were more likely to have sufficient S-25(OH)D concentration (≥50 nmol/L) than those who drank milk less frequently (odds ratio 2.4, 95% CI 1.7–3.3) [47,48]. A Spanish study in 9–13-year-old children (*n* = 102) showed that the number of daily dairy servings (mean 2.3 servings) was associated with the S-25(OH)D status [49]. Those who consumed ≥2.5 servings of milk daily had higher S-25(OH)D concentrations than those who did not (53 nmol/L vs. 46 nmol/L) [49]. In Sweden, fortified lean milk consumption (mean 230 g/day) correlated with S-25(OH)D status in a group of 13-year-old children (*n* = 165) [50]. Among 15–18-year-old adolescents in Norway (*n* = 890), the use of vitamin D-fortified milk was significantly associated with S-25(OH)D status in a multivariate model in boys, but not in girls [51]. Boys who were frequent milk consumers had higher S-25(OH)D concentrations than infrequent consumers, but this was not seen among girls, and no milk consumption data was provided [51].

#### 3.2.2. Pregnant Women and Mother-Child Pairs

Two studies carried out in pregnant women in Thailand and Finland also showed an association between vitamin D-fortified milk and vitamin D status [52,53]. Among Thai women (*n* = 120), the consumption of multivitamin-fortified milk containing vitamin D was higher among those with vitamin D sufficiency (S-25(OH)D concentration > 50 nmol/L) in the third trimester than among those with insufficiency [52]. Consumption of multivitamin-fortified milk was associated with an increase in S-25(OH)D concentration between the first and third trimester. The mean multivitamin-fortified milk consumption was 1.0 daily serving in the first trimester and 1.4 servings in the third trimester. In a multivariate analysis, non-consumption of multivitamin-fortified milk was an independent predictor of vitamin D deficiency [52]. In a study carried out in 584 mother-child pairs in Finland, modifiers of umbilical cord blood (UCB) 25(OH)D status were studied [53]. The maternal dietary pattern “dairy and sandwich”, including vitamin D-fortified milk and margarines, positively contributed to child UCB 25(OH)D status in mother-child pairs in whom an increase was seen in 25(OH)D concentration when comparing maternal 25(OH)D status in early pregnancy with UCB 25(OH)D status, but not in those in whom no increase was seen [53]. Among Jordanian women, no differences in S-25(OH)D concentration were seen among those who consumed vitamin D-fortified milk relative to those who consumed unfortified milk (26 nmol/L vs. 27 nmol/L) [44]. However, their vitamin D status was much worse than that of their children, whose vitamin D status was better if vitamin D-fortified milk was consumed [44].

#### 3.2.3. Adults, the Elderly, and All Age Groups

Among adults aged 20–65 years in the United States (*n* = 743), the use of vitamin D-fortified milk was a significant predictor of S-25(OH)D status in the wintertime, but not in the summertime [39]. In an elderly American population aged >65 years (*n* = 376) [41], S-25(OH)D status correlated with milk calcium intake from vitamin D-fortified milk (an indicator of milk consumption). Among those who did not use vitamin D supplements, milk calcium was the main determinant of S-25(OH)D status; however, this was not the case among vitamin D supplement users. Among adult (≥18 years) Arab-American women (*n* = 87), vitamin D-fortified milk was not an independent determinant of S-25(OH)D status, but their milk consumption was minimal, and S-25(OH)D concentrations were extremely low [54]. In the Canadian National Survey consisting of a population aged 6–79 years (*n* = 5306), those who consumed vitamin D-fortified milk once a day or more had higher S-25(OH)D concentrations than those with consumption of less than once a day [55]. People who consumed milk more than once a day had a mean S-25(OH)D concentration of 75 nmol/L, while the corresponding mean among those who consumed milk less than once a day was 63 nmol/L. In a Swedish study on elderly women aged >60 years (*n* = 116), consumption of vitamin D-fortified reduced-fat dairy correlated with S-25(OH)D status, and the intake of two daily portions of fortified milk (300 g) was associated with a 6.2 nmol/L increment in S-25(OH)D concentration in a multiple linear regression model [56]. Further, in an Irish study of three large cohorts of elderly subjects (*n* = 1233, *n* = 1895, *n* = 1316), vitamin D-fortified milk consumption predicted a higher S-25(OH)D concentration in two of the three cohorts [57].

## 4. Discussion

Based on these observational studies on vitamin D intake and vitamin D-fortified milk consumption, it seems that in countries with wide vitamin D fortification policies (Finland, Canada, United States), the total vitamin D intake as well as the contribution of milk to total vitamin D intake is higher than in the countries without fortification policies (Ireland, United Kingdom, Spain, Australia). It is notable that in Norway and Sweden, where some of the fluid milks are fortified with vitamin D amounts lower than in Finland, Canada, or United States, the contribution of fluid milk to vitamin D intake was shown to be as low as 4% and 12%, respectively, compared with around 50% in the other fortification policy countries.

Concerning the vitamin D status, we observed that the consumption of vitamin D-fortified milk was positively associated with 25(OH)D status in almost all studies included in this review within heterogeneous population groups, independent of country-specific vitamin D-fortification policies. Even though the consumed amounts of milk varied, the associations between milk and 25(OH)D status were seen also at fairly low consumption levels. Further, the association was seen in different population groups: children (with the exception of 10-year old Finns), teenagers (except in Norwegian girls), adults (except in Arab-American women), pregnant women, and the elderly. This mostly positive association between vitamin D-fortified milk consumption and vitamin D status was supported by a recent standardized representative population-based study in Finland, where the vitamin D fortification policy of fluid milks, in particular, was shown to be successful in improving vitamin D status in the Finnish population [25]. It would be useful to have systematic follow-up data from the other countries with vitamin D fortification policies, as the present evidence is based mainly on the Finnish follow-up study. Vatanparast et al. [35] stated that despite vitamin D fortification being mandatory in Canada, the vitamin D intakes are inadequate and recently, Canada implemented new guidelines to increase the fortification levels [11]. Sweden has also extended their vitamin D fortification policy [10]; thus, in the following years there is an opportunity to evaluate the effects of vitamin D fortification at the population level also in those countries.

### 4.1. Limitations of the Study

The studies included in this review were carried out in populations of differing size, age, and gender in numerous countries and at a range of latitudes with different levels of UVB exposure. Different assay methods for 25(OH)D analysis have been used, increasing the heterogeneity of the studies [58], and not all studies have provided quality control data. LC-MS/MS, which is considered the golden standard and reference method in 25(OH)D assays [58], was used in 25% of the reviewed studies. However, as our aim was to investigate the associations between the consumption of vitamin D fortified milk and 25(OH)D status, the differences among the assays probably do not mitigate the power of the overall conclusions, as the trends in 25(OH)D concentrations usually remain similar independent of the analysis method used [59]. Of greater importance is that most studies considered the variability of 25(OH)D concentrations between seasons and took the samples at a time when UVB availability is low or over a short time period or adjusted the data for the season [39,56]. Also, the dietary assessment methods used in the studies varied and the validity of the methods was not described in all papers. Some used validated FFQs [25,47] and some only used questionnaires on milk consumption [43,46]. Moreover, the portion sizes used were not defined in all studies. The consumed amounts of milk and the vitamin D contents differed, as did the confounding factors used in statistical analyses. The representativeness of the samples was not described in most of the studies, but representative data from national health surveys in the United States, Canada, Sweden, Norway, and Finland were included when describing the contributions of fluid milk to vitamin D intake [25,27,28,33,34,35,37]. We only searched data from PubMed, and some studies might have been missed in our limited literature search. However, these are probably studies that have not taken a stand on vitamin D fortification of fluid milks as such, and therefore, have not emphasized it in the abstracts or in the keywords. Nevertheless, publication bias may have occurred, as some studies that found no association between vitamin D-fortified milk and vitamin D status may not have been published.

### 4.2. Future Perspectives in Vitamin D Fortification 

Vitamin D fortification of foodstuffs has proven to be a suitable vehicle to increase vitamin D intake at the population level [5], and the present review shows that vitamin D-fortified fluid milk products contribute to both vitamin D intake and 25(OH)D status. Cashman and Kiely [5], however, stated that the fortification of fluid milks may not be enough. Thus, country-specific staple foods should be chosen as optimal vitamin D carriers based on the results of simulation studies. Also, the biofortification of foodstuffs should be considered [5]. In many countries without a current fortification policy, the option of systematic vitamin D fortification of food is under consideration, and simulation studies have been carried out in recent years. A study using Swedish, British, and Dutch data, for instance, showed that increased fortification of fluid milk to the level currently used in Finland (1 µg/100 g) and fortification of margarines to 15 µg/100 g would substantially increase vitamin D intake [60]. Another study based on British data [61] revealed that the best option would be the fortification of wheat flour with vitamin D, this being a more efficient option to increase S-25(OH)D concentration than milk alone or combined fortification of milk and wheat flour. In Germany, the effects of fortification on the seasonal variation of S-25(OH)D concentrations were simulated, but milk was not considered to be a good carrier of vitamin D [62]. Simulation studies in Irish and British children showed that the fortification of cow’s milk would improve vitamin D intake [14,63]. Further, an Australian simulation demonstrated that with vitamin D fortification of all milk and breakfast cereals, vitamin D intake would increase almost two-fold [17]. These studies reflect the interest in widening the fortification policies. However, the results of the above-described simulation studies show that fortification of milk products may not be the most effective option in all countries.

## 5. Conclusions

The reviewed studies indicated that in countries with a national vitamin D fortification policy for fluid milks at a level of around 1 µg/100 g, such as Finland, United States, and Canada, milk products contribute substantially to vitamin D intake, while in countries without a fortification policy or with only a few milk products being mandatorily fortified, the contribution is low. Studies carried out at different latitudes among different population groups have also shown that the consumption of vitamin D-fortified milk is associated with a higher 25(OH)D concentration. Based on the reviewed observational studies, vitamin D fortification of milk is an effective vehicle in improving vitamin D intake and 25(OH)D status in populations with adequate average milk consumption. However, other food sources, natural or fortified, as well as national recommendations on the use of vitamin D supplements should not be overlooked when planning national nutrition policies to ensure adequate vitamin D intake.

## Figures and Tables

**Table 1:** Countries with a vitamin D fortification policy of fluid milk products.

Country	Vitamin D-Fortified Milk Products	Type of Fortification	Added Amount of Vitamin D	New Proposed Amounts of Vitamin D
Finland [6,7]	fluid milk products (milk, yoghurt, sourmilk) *	voluntary	1 µg/100 g	na
Norway [8]	extra low-fat milk (also lactose free)	voluntary	0.4 µg/100 g	na
Sweden [9,10]	low-fat milk (max 1.5% fat)	mandatory	0.38–0.50 µg/100 g	0.95–1.10 µg/100 g for milk <3% fat 0.75–1.10 µg/100 g for fermented milk <3% fat
Canada [11]	milk	mandatory	0.825–1.125 µg/100 g	2 µg/100 g
United States [12]	fluid milk (also acidified milk and cultured milk), yoghurt	voluntary ^‡^	1.05 µg/100 g for milk 2.225 µg/100 g for yoghurt ^§^	na

* In regard to organic milk products, it is mandatory to add 1 µg/100 g vitamin D to homogenized fat-free milk (not allowed on other organic milk products). ^‡^ for milk products, only evaporated and non-fat dry milk are mandatorily fortified. ^§^ maximum amount; na = not applicable.

**Table 2:** Studies on the contribution of milk to total or dietary vitamin D intake.

Reference	Country	Study Population	Dietary Assessment Method	Total/Dietary Vitamin D Intake (µg/day)	SD(or SEM *)	Vitamin D Intake from (fortified) Fluid Milk or Related Products (µg/day)	SD	Contribution of (Fortified) Milk to Total or Dietary Vitamin D Intake (%)
Children and adolescents								
Black et al. (2014) [13]	Ireland	594 children, 5–12 years and 441 teenagers, 13–17 years	7-day (semi-) weighted food record	Total/dietary intake 5–8 years: 2.8/1.9 9–12 years: 2.8/2.2 13–17 years: 3.2/2.6	2.4/1.1 2.1/1.3 2.5/1.8	Fortified milk: 0.1 Milk and yoghurt: 0.3–0.4	na	Total intake Fortified milk: 2–3% Milk and yoghurt: 10–13%
Cole et al. (2010) [29]	United States	290 children, 1–5 years	3-day food record	Dietary intake: 4.4	3.0	Fortified milk: 2.7 ^†^	na	Dietary intake Fortified milk: 62%
Cribb et al. (2015) [14]	United Kingdom	755 children, 1.5 years and 3.5 years	3-day food diary	Dietary intake 1.5 years: 1.6 3.5 years: 1.8	1.5 1.4	Yoghurt, cheese and milk 1.5 years: 0.035 µg/MJ/day 3.5 years: 0.023 µg/MJ/day	0.02 µg/MJ/day 0.02 µg/MJ/day	Dietary intake Yoghurt, cheese and milk 1.5 years: 9% 3.5 years: 6%
Hennessy et al. (2016) [15]	Ireland	500 children, 1–4 years	4-day weighted food diary	Total intake All subjects: 3.5 Fortified food consumers: 3.2	3.7 2.7	Fortified milk All subjects: 0.1 Fortified food consumers: 0.1	na	Total intake All subjects: 2% Fortified food consumers, supplement non-users: 13%
Mark et al. (2011) [30]	Canada	159 children, 8–11 years	3 × 24 h recalls	Total/dietary intake: 6.6/5.6	4.3/3.5	Milk: 3.3 ^†^	na	Total/dietary intake Milk: 49/58%
Piirainen et al. (2007) [32]	Finland	36 children, 4 years	4-day food record	Total/dietary intake: 7.9/4.5	6.3–9.6/3.8–5.1 ^§^	2.3	2.0–2.6 ^§^	Total intake Milk: 54%
Soininen et al. (2016) [31]	Finland	374 children, 6–8 years	4-day food record	Total/dietary intake: 7.7/5.9	na/2.1	Fluid milk: 2.9 All milk products: 3.1	1.5 1.4	Total/dietary intake Fluid milk: 38/49% All milk products: 40/52%
Adults and the elderly								
Amcoff et al. (2012) [28]	Sweden	1797 adults, 18–80 years	4-day food diary	Dietary intake Women: 6.4 Men: 7.6	4.2/5.4	na	na	Dietary intake Milk products: 12%
Gonzalez-Rodriguez et al. (2013) [16]	Spain	418 adults, 18–60 years	24 h recall	Total/dietary intake: 3.5/3.2	4.0/3.8	Dairy products: 0.5 ^†^	na	Total/dietary intake Dairy products: 15/17%
Holm Totland et al. (2012) [27]	Norway	1787 adults, 18–70 years	24 h recall	Total/dietary intake Women: 10/4.9 Men: 12/6.7	na/4.3 na/5.7	na	na	Dietary intake Vitamin D fortified extra-skimmed milk: 4%
Jayaratne et al. (2013) [17]	Australia	785 adults, ≥31 years	FFQ	Total intake: 4.4	4.0	Dairy and related products including margarine: 1.9 ^†^ Milk: 0.8 ^†^ Yoghurt: 0.3 ^†^	na na na	Total intake Dairy and related products including margarine: 43% Milk: 18% Yoghurt: 6%
Jääskeläinen et al. (2017) [25]	Finland	3635 adults, ≥30 years	FFQ	Dietary intake Men: 14 Women: 12	14–15 ^§^ 11–12 ^§^	na	na	Dietary intake Fluid milk products: 34%
Kinyamu et al. (1998) [41]	United States	376 elderly women, 65–77 years	7-day food record	Total intake Supplement non-users: 3.5 Supplement users: 13.4	2.2 2.0	Milk Supplement non-users: 2.0 Supplement users: 1.8	1.6 1.5	Total intake Milk: 51%
Kolahdooz et al. (2013) [38]	Canada	203 Inuit and Inuvialuit women, 19–44 years	FFQ	All subjects: 6.0 ^‡^ Traditional food eaters: 7.1 ^‡^ Non-traditional food eaters: 4.9 ^‡^	6.3 5.3 3.2	Dairy group (milk, yoghurt, cheese and eggs) Traditional food eaters: 2.2 Non-traditional food eaters: 1.9	na na	Dairy group (milk, yoghurt, cheese and eggs) Traditional food eaters: 31% ^‡^ Non-traditional food eaters: 39% ^‡^
Levy et al. (2015) [39]	United States	743 adults, 20–65 years	one week diet history	Total intake Winter season: 4.5 Summer season: 4.3	4.0 3.2	Dairy products Winter season: 1.9 Summer season: 1.9	2.5 3.8	Dietary intake Winter season: 43% Summer season: 41%
Moore et al. (2014) [37]	United States	9719 adults, ≥19 years	24 h recall	Total/dietary intake 8.6/4.4	0.3/0.1 *	Milk and milk drinks: 1.7 ^†^ Fortified milk and milk products: 1.9 ^†^	na na	Total/dietary intake Milk and milk drinks: 20/39% Fortified milk and milk products: 22/44%
O’Dowd et al. (1993) [41]	United States	109 elderly, >60 years	FFQ or 3-day dietary record	Total/dietary intake All subjects: 9.5/ Supplement non-users: 7.3	5.1/2.5	Fortified milk All subjects: 4.7	1.9	Total intake Fortified milk: 50%
Poliquin et al. (2009) [33]	Canada	9425 adults, ≥25 years	interview-administered semi-quantitative FFQ	Total intake from milk and supplements Women: 5.6 Men: 4.8	5.9 5.5	Milk Women: 2.7 Men: 3.0	2.9 3.5	Total intake from milk and supplements Women: 48% Men: 63%
Raulio et al. (2017) [42]	Finland	1295 adults, 25–64 years	24 h recall	Total intake Women: all women: 17.5 Supplement non-users: 8.6 Supplement users: 24.7 Men: all men: 17.3 Supplement non-users: 11.2 Supplement users: 29.5	15.4 6.2 16.8 17.0 7.5 23.1	na	na	Dietary intake Milk: 28–39%, depending on age and sex
All ages								
Hill et al. (2012) [36]	United States and Canada	7837 US and 4025 Canadian citizens, ≥2 years	7- to 14-day household food diary	Total intake United States: 4.4 Canada: 4.2	0.03 * 0.5 *	Milk United States: 2.0 Canada: 1.9	na na	Total intake Milk: 44% in both countries
Moore et al. (2004) [34]	United States	18931 subjects, >1 years	24 h recall	Total/dietary intake: 5.3–9.8/3.9–7.0 depending on age and sex	na	na	na	Dietary intake Dairy products: 45–47%
Vatanparast et al. (2010) [35]	Canada	34789 subjects, >1 years	24 h recall	Dietary intake: 6.2	0.1 *	Milk products: 2.9	na	Dietary intake Milk products: 49%

FFQ, food frequency questionnaire; na, not applicable; * SE(M), standard error (of mean); ^†^ calculated from the proportion of milk contribution; ^‡^ unclear whether total or dietary vitamin D intake (3% supplement users); ^§^ 95% confidence interval.

**Table 3:** Studies in which the contribution of vitamin D-fortified milk to serum or plasma 25-hydroxyvitamin D status was evaluated.

Reference	Country (Latitude)	Season Blood Drawn	Study Population	25(OH)D Assay Method (Quality Control of Assay: Certificate; CV% <15%)	Dietary Assessment Method	Serum or Plasma 25(OH)D nmol/L Mean (or Median *)	SD (or IQR ^†^ or 95% CI ^‡^ or SE ^§^)
Children and adolescents							
Abu Shady et al. (2016) [43]	Egypt (31° N)	April, May	200 children, 9–11 years	Quantitative enzyme immunoassay (na; na)	Questionnaire	41	14
Barman et al. (2015) [50]	Sweden (63° N)	All	165 children, 13 years	LC-MS/MS (na; na)	FFQ	51	14
Cole et al. (2010) [29]	United States (33° N)	All	290 children, 1–5 years	LC-MS/MS (na; na)	3-day food record	65	19
Lee et al. (2014) [46]	Canada (43° N)	All	2468 children, 1–6 years	Diasorin LIAISON (na; yes except inter CV% 17.4% at high concentrations)	Questionnaire	80 *	66–99 ^†^
Mark et al. (2011) [30]	Canada (45° N)	All	159 children, 8–11 years	IDS radioimmunoassay (na; yes)	3 × 24 h recalls	Winter/spring: 50 Summer/autumn: 58	10 15
Munasinghe et al. (2017) [47,48]	Canada (various latitudes)	All	2270 children, 3–18 years	Diasorin LIAISON (na; yes)	FFQ	62	56–69 ^‡^
Rodríguez–Rodríguez et al. (2011) [49]	Spain (40° N)	February	102 children, 9–13 years	Chemiluminescence (na; na)	3-day weighted food diary	50	16
Rosendahl et al. (2017) [45]	Finland (60° N)	January–June	171 children, 10 years	Roche Diagnostics immunocheminuminescence (na; na)	FFQ	73	22
Soininen et al. (2016) [31]	Finland (62° N)	All but July	374 children, 6–8 years	Diasorin LIAISON (na; yes)	4-day food record	69	24
Öberg et al. (2014) [51]	Norway (69° N)	September–April	890 children, 15–18 years	LC–MS/MS (DEQAS; yes)	Questionnaire	Boys: 41 Girls: 54	21 23
Pregnant women and mother-child pairs							
Charatcharoenwitthaya et al. (2013) [52]	Thailand (14° N)	Winter season: 72%, rainy season: 28%	120 pregnant women, 18–40 years	LC–MS/MS MassCrom (na; yes)	Interviewed questionnaire	1st trimester: 61 2nd trimester: 84 3rd trimester: 90	17 20 22
Gharaibeh et al. (2009) [44]	Jordan (31° N)	June and July	93 children (4–5 years) and mothers (mean age 34 years) dyads	IDS ELISA (na; na)	Questionnaire	Mothers: 26 Children: 56	10 20
Hauta–alus et al. (2017) [53]	Finland (60° N)	All	584 newborns and mothers (18–43 years)	IDS–iSYS (DEQAS; yes)	FFQ	Mothers: 89 Cord blood: 88	19 22
Adults and the elderly							
Burgaz et al. (2007) [56]	Sweden (60° N)	January–March	116 elderly women, 61–86 years	IDS EIA (na; yes)	FFQ	69	23
Hobbs et al. (2009) [54]	United States (42° N)	April	87 women, ≥18 years	Diasorin LIAISON (na; na)	Questionnaire	Unveiled subjects: 21 * Veiled supplement users: 17 * Veiled supplement non-users: 10 *	14–34 ^†^ 10–29 ^†^ 5–17 ^†^
Kinyamu et al. (1998) [41]	United States (41° N)	All	376 elderly women, 65–77 years	Competitive binding assay (na; yes)	7-day food record	Supplement non-users: 74 Supplement users: 88	23 28
Levy et al. (2015) [39]	United States (various latitudes)	February–April and August–October	743 adults, 20–65 years	Diasorin LIAISON (College of American Pathology; na)	One week diet history	Summer: 101 Winter: 93	42 39
McCarroll et al. (2015) [57]	Ireland (52° N)	All	3 cohorts (1233, 1895, 1316) of elderly subjects, >60 years	LC–MS (DEQAS; yes)	Questionnaire	Supplement non-users: 46/61/68 Supplement users: 67/83/74	24/32/23 27/27/30
O’Dowd et al. (1993) [40]	United States (41° N)	January–May	109 elderly, >60 years	Competitive binding assay (na; yes)	FFQ or 3-day dietary record	All subjects: 45 Supplement non-users: 40 Supplement users: 65	2 ^§^ 2 ^§^ 3 ^§^
All ages							
Langlois et al. (2010) [55]	Canada (various latitudes)	All	5306 subjects, 6–79 years	Diasorin Liaison (DEQAS; yes)	Interviewed questionnaire	All subjects 68 April–October 70 November–March 64	65–70 ^‡^ 66–74 ^‡^ 60–68 ^‡^

CI, confidence interval; CV coefficient of variation; DEQAS, Vitamin D External Quality Assessment Scheme; EIA enzyme immunoassay; ELISA enzyme linked immunosorbent assay; FFQ, food frequency questionnaire; IQR interquartile range; LC-MS/MS liquid chromatography-tandem mass spectrometry; SE standard error; 25(OH)D, 25-hydroxyvitamin D. * Median; ^†^ IQR; ^‡^ 95% CI; ^§^ SE.
